# Genomic and transcriptomic analysis of the endophytic fungus *Pestalotiopsis fici* reveals its lifestyle and high potential for synthesis of natural products

**DOI:** 10.1186/s12864-014-1190-9

**Published:** 2015-01-27

**Authors:** Xiuna Wang, Xiaoling Zhang, Ling Liu, Meichun Xiang, Wenzhao Wang, Xiang Sun, Yongsheng Che, Liangdong Guo, Gang Liu, Liyun Guo, Chengshu Wang, Wen-Bing Yin, Marc Stadler, Xinyu Zhang, Xingzhong Liu

**Affiliations:** State Key Laboratory of Mycology, Institute of Microbiology, Chinese Academy of Sciences, Beijing, China; Department of Plant Pathology, China Agricultural University, Beijing, China; Department of Natural Products Chemistry, Beijing Institute of Pharmacology & Toxicology, Beijing, China; Key Laboratory of Insect Development and Evolutionary Biology, Institute of Plant Physiology and Ecology, Shanghai Institutes for Biological Sciences, Chinese Academy of Sciences, Shanghai, China; Department Microbial Drugs, Helmholtz Centre for Infection Research, Braunschweig, Germany

**Keywords:** Genome, Endophyte, *Pestalotiopsis fici*, Secondary metabolite

## Abstract

**Background:**

In recent years, the genus *Pestalotiopsis* is receiving increasing attention, not only because of its economic impact as a plant pathogen but also as a commonly isolated endophyte which is an important source of bioactive natural products. *Pestalotiopsis fici* Steyaert W106-1/CGMCC3.15140 as an endophyte of tea produces numerous novel secondary metabolites, including chloropupukeananin, a derivative of chlorinated pupukeanane that is first discovered in fungi. Some of them might be important as the drug leads for future pharmaceutics.

**Results:**

Here, we report the genome sequence of the endophytic fungus of tea *Pestalotiopsis fici* W106-1/CGMCC3.15140. The abundant carbohydrate-active enzymes especially significantly expanding pectinases allow the fungus to utilize the limited intercellular nutrients within the host plants, suggesting adaptation of the fungus to endophytic lifestyle. The *P. fici* genome encodes a rich set of secondary metabolite synthesis genes, including 27 polyketide synthases (PKSs), 12 non-ribosomal peptide synthases (NRPSs), five dimethylallyl tryptophan synthases, four putative PKS-like enzymes, 15 putative NRPS-like enzymes, 15 terpenoid synthases, seven terpenoid cyclases, seven fatty-acid synthases, and five hybrids of PKS-NRPS. The majority of these core enzymes distributed into 74 secondary metabolite clusters. The putative Diels-Alderase genes have undergone expansion.

**Conclusion:**

The significant expansion of pectinase encoding genes provides essential insight in the life strategy of endophytes, and richness of gene clusters for secondary metabolites reveals high potential of natural products of endophytic fungi.

**Electronic supplementary material:**

The online version of this article (doi:10.1186/s12864-014-1190-9) contains supplementary material, which is available to authorized users.

## Background

Endophytic fungi live within healthy plants without causing any apparent symptoms of disease [1]. In natural ecosystems, endophytic fungi have been isolated from almost all plants studied so far. They confer abiotic and biotic stress tolerance, increase biomass, and decrease water consumption of the host plant [2]. In recent years, they have been received increasing attention from natural product chemists due to their various novel and bioactive compounds [3-7]. The functions of bioactive natural products include antibiotics, anticancer agents, agrichemicals, and other bioactive compounds [5]. Some of them could be developed into leads for therapeutics, such as the well-known taxol [8]. In addition, fungal endophyte is also proposed to be potential source of biocatalysts [9]. Endophytes as important biological resources are waiting to be exploited.

The genus *Pestalotiopsis* (Xylariales, Ascomycota) includes many widely distributed species, occurring on a wide range of substrata such as on living plants as pathogens and endophytes and on dead plant materials as saprobes [10]. However, *Pestalotiopsis* spp. have been extensively isolated from healthy plant tissues and considered as a main part of endophytes in the past decade [11-13]. The chemical investigations showed that *Pestalotiopsis* spp. are important resource for natural product discovery [14,15].

*Pestalotiopsis fici* Steyaert was first identified as a pathogen of *Ficus carica* [16]. However, a strain of *P. fici* (W106-1/CGMCC3.15140) was isolated as endophyte from the branches of *Camellia sinensis* in Hangzhou, China. Chemical investigations revealed that this strain produces 88 secondary metabolites including 70 new natural products [17]. Those include, for instance, pestaloficiols A-L and Q-S [18-20], pestalofones A-H [21,22], pestalodiols A-D [22], chloropupukeananin which is the first chlorinated pupukeanane derivative discovered in fungi [23], chloropestolides A-G with unprecedented spiroketal skeleton [24,25], chloropupukeanone A [26], chloropupukeanolides A-E [26,27]. Those compounds have shown various bioactivities, including inhibition of HIV-1 replication, cytotoxicity against human tumor cell lines, and antifungal effects against *Aspergillus fumigatus* [18-22,24-27]. It has been hypothesized that the biosynthesis pathways for some of these secondary metabolites include a Diels-Alder reaction, which is vital for the observed abundance of secondary metabolites [17]. Although putative biosynthesis pathways of some secondary metabolites are postulated, the actual biosynthetic pathways remain to be confirmed. However, access to the genes involved in secondary metabolism has been greatly enhanced, as the putative genes encoding for biosynthesis of secondary metabolites can easily be detected by *in silico* analysis of genomic data [28-30].

Both lifestyle and richness of secondary metabolites of endophytic fungi have not been comprehensive understood. In this study, the *P. fici* genome was sequenced and annotated. The gene families encoding carbohydrate-active enzymes especially pectinases and transporters have undergone expansion. A large set of genes involved in secondary metabolism has been identified. The genomic information provides insight on how the living strategy as endophyte and how the richness and diversity of secondary metabolites.

## Results

### Tea branch colonization by *Pestalotiopsis fici*

**Figure 1:**
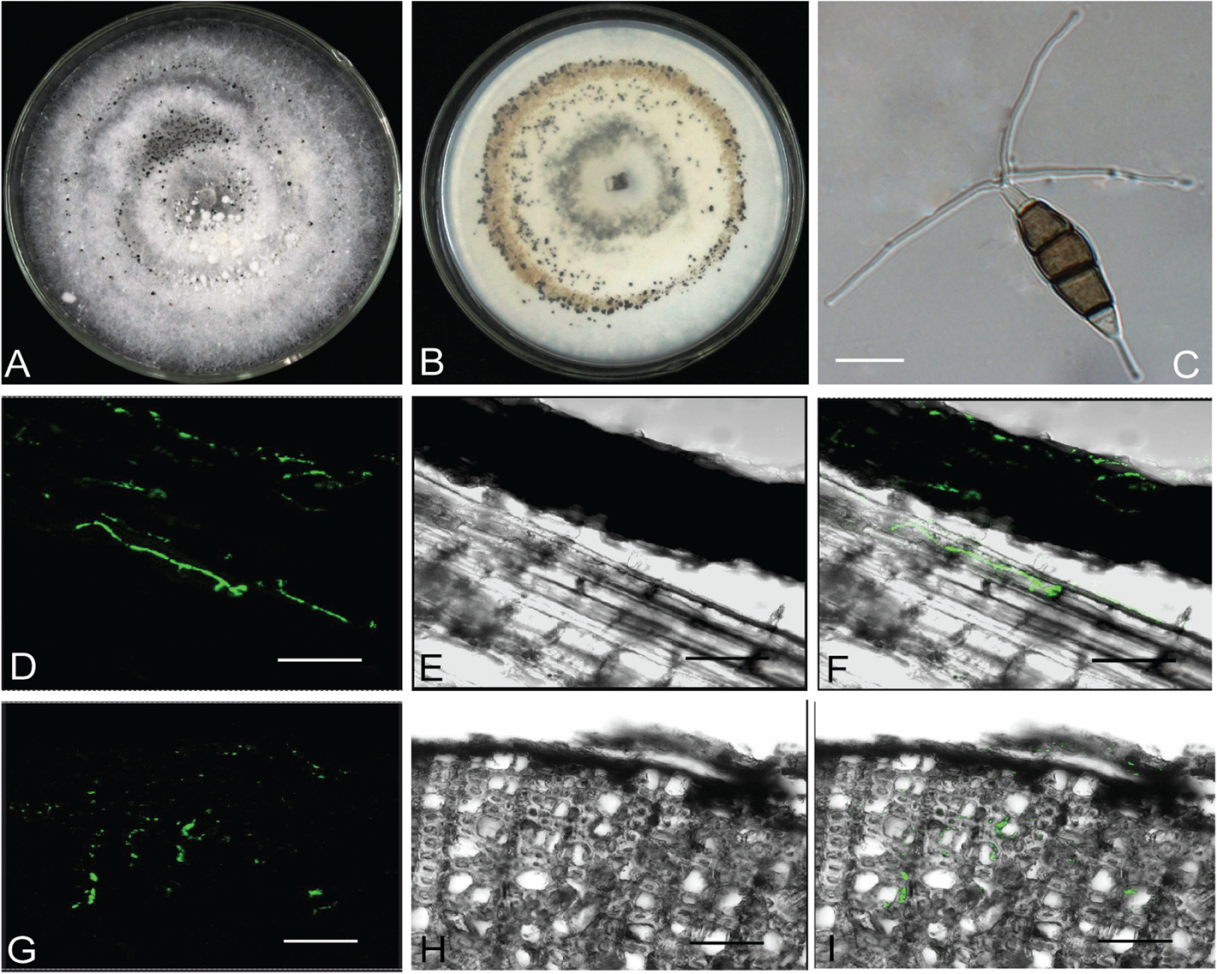
**Morphological characteristics of** ***Pestalotiopsis fici*** **and its biotrophic growth in a tea branch. A)** and **B)**, Culture on PDA; **C)** Typical conidia; **D – F)** Longitudinal sections of a tea branch 7 days after inoculation with *P. fici* hyphae; **D)** Fluorescent micrograph of tea and hyphae; **E)** Brightfield micrograph of **D)**; **F)** Overlay of fluorescent and brightfield micrographs; **G – I)** Cross sections of a tea branch 7 days after inoculation with *P. fici* hyphae; **G)** Tea branch and hyphae; **H)** Brightfield micrograph of **G)**; **I)** Overlay of fluorescent and brightfield micrographs. Scale bar = 10 μm in C and 50 μm in **D** – **I**.

Although *P. fici* was isolated as endophyte from the tea plant, the detailed knowledge about fungal colonization strategy is unknown. The twigs of the tea tree were inoculated with fresh mycelium of the GFP transformant of *P. fici* (GFP3-1) and the colonization pattern was documented over a period of 21 days by confocal microscopy. A few hyphae were observed at seven (Figure 1) and 21 days (Additional file 1: Figure S1) after inoculation respectively, in the living tea twigs without any disease symptoms.

### General genome features

The *P. fici* genome was assembled into 118 scaffolds (24.5-fold coverage) with N50 of 4 Mb encompassing 52 Mb (Table 1). A total of 15,413 genes were predicted, including 11,755 orthologous genes and 14,528 genes containing at least one domain/motif (Additional file 1: Figure S2). Among them, 494 genes were pseudogenes. Repetitive sequences, including 0.49% simple repeats, 0.96% low complexity repeats, and 1.54% transposable elements (TEs), made up only 2.97% of the genome of *P. fici*. The TEs were identified, grouped, and annotated as class 1 (LTR, LINE), class 2 (MITE, TIR) or unknown TEs using the REPET pipeline and Repbase. The LTR group in class 1 comprised of two families: Gypsy and Copia. RIPCAL analysis showed index values of 0.35 for (CpA+TpG)/TpA and 0.42 for (CpT+ApG)/(TpT+ApA), which suggested heavy repeat-induced point mutation (RIP) in the *P. fici* genome and that the RIP mutation was a classical pattern of CpA→TpA (Additional file 1: Figure S3).

**Table 1:** **Main features of the** ***Pestalotiopsis fici*** **genome**

**Feature**	***P. fici***
Assembly size (Mb)	52
Scaffold N50 (Mb)	4
Coverage (fold)	24.5
GC content (%)	48.73
GC exon (%)	53.56
GC intron (%)	44.18
Repeat content (%)	2.97
Protein-coding genes	15,413
Gene density (genes per Mb)	296.9
Exons per gene	2.76
tRNA genes	258
rRNA genes	52
Pseudogenes	494
Transposable elements (%)	1.54

Mb: mega base pairs.

**Figure 2:**
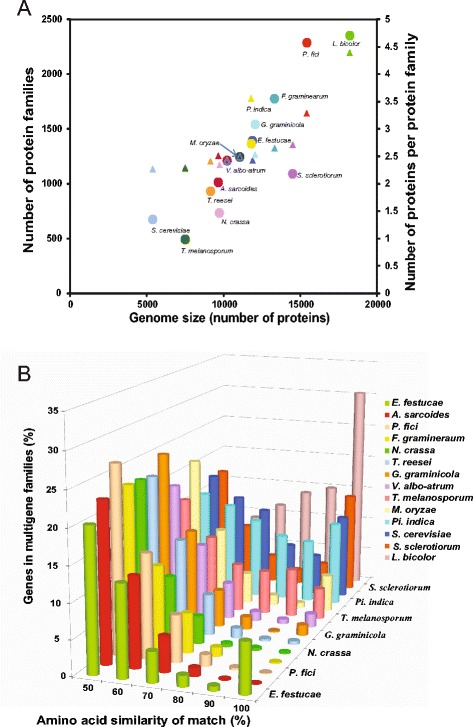
***Pestalotiopsis fici*** **possesses a high proportion of genes in multigene families and few highly similar genes. A)** Relationship between genome size and number of protein families and proteins per family, circle indicates the number of protein families and a triangle indicates the number of proteins per family; **B)** Histogram of amino acid percent identity of top-scoring self-matches for genes in *P. fici* and selected sequenced eukaryotic genomes. For each fungus, the protein and coding regions for each gene were compared with those of every other gene in the same genome using BLASTX.

One of the most novel characteristics of the *P. fici* genome was that it contained more multigene families, compared with those of other reference ascomyceteous fungi in this study. The multigene families in the *P. fici* genome are 2,047 that are similar to that in the genome of the ectomycorrhizal basidiomycete, *Laccaria bicolor* (Figure 2A and Additional file 1: Figure S4). The average number of proteins per family in *P. fici* (3.29) was much higher than in other Pezizomycotina species (2.46) but was similar to the endophytic basidiomycete, *Piriformospora indica* (3.56) (Figure 2A). The *P. fici* genome, however, contained a large number of replicated gene pairs with amino acid identities below 80% (Figure 2B).

**Figure 3:**
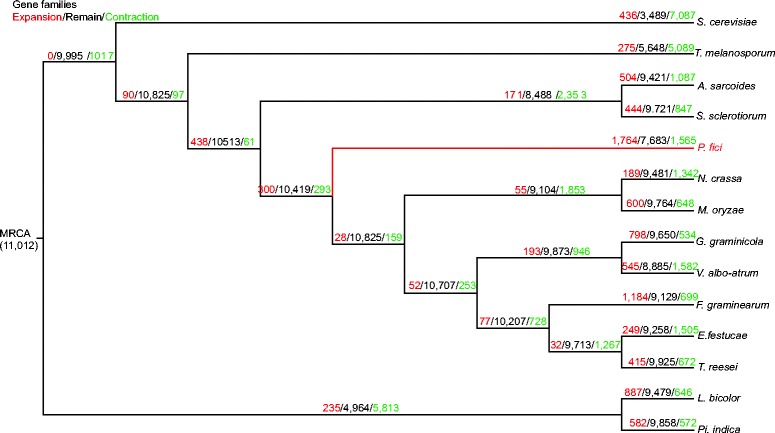
**Gene families expansion and contraction in the genomes of** ***Pestalotiopsis fici*** **and selected representative fungi as predicted by CAFÉ.** The number of gene families that have undergone expansion/remained the same/contraction are indicated in red/black/green, respectively.

CAFÉ analysis revealed that 1,764 families had expanded in the *P. fici* genome (Figure 3), indicated a considerable protein family expansion. The number of expanded gene family was significantly higher for *P. fici* than that of the reference fungi. Gene family expansion occurred in those genes encoding for cytochrome P450 monooxygenases (CYPs), heterokaryon incompatibility, major facilitator superfamily (MFS), short-chain dehydrogenase, tyrosinase, intradiol ring-cleavage dioxygenase, methyltransferase type, and cysteine-rich fungal-specific extracellular EGF-like (CFEM) domain-containing protein (Additional file 1: Figure S5 and Additional file 2: Table S2). The expanded gene families of the *P. fici* genome seem to be mainly involved in processes like secondary metabolism, pheromone response, detoxification, and virulence (Additional file 1: Figure S5).

### Carbohydrate-active enzymes (CAZymes) in *P. fici*

**Figure 4:**
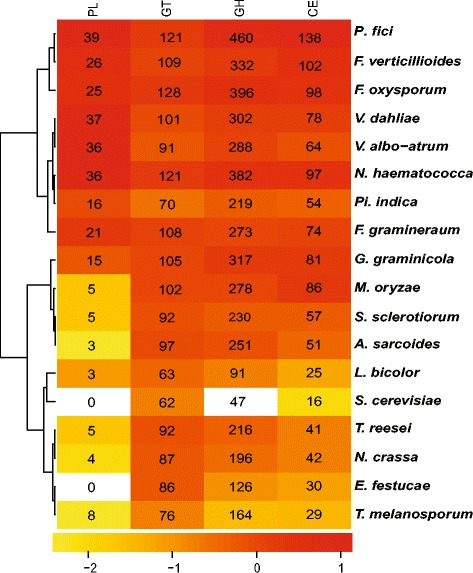
**Hierarchical clustering of CAZyme classes from** ***Pestalotiopsis fici*** **and 16 other fungal genomes.** The numbers of enzyme modules in each genome are indicated and the background color changes from white to red are depicted as binary logarithm of folds (−2, −1, 0, 1 in the below of the figure) of each CAZyme family gene numbers of each genome divided average each CAZyme family gene numbers of all genomes analyzed. CAZyme categories included glycoside hydrolase (GH), glycosyl transferase (GT), polysaccharide lyase (PL) and carbohydrate esterase (CE).

Fungi can utilize monosaccharides or oligosaccharides, which were degraded from polysaccharides by secreting a variety of CAZymes. *P. fici* has the highest number of putative CAZymes genes (Figure 4) and the most abundant CAZyme families (Additional file 1: Figure S6 and Additional file 2: Table S3), compared with those of 17 other genome-sequenced fungi (Listed in Additional file 2: Table S1), followed by parasites, saprophytes, and symbionts. These expanded CAZyme arsenals of *P. fici* are similar to those of *Fusarium oxysporum* and *F. verticillioides*, and the total CAZyme repertoire for *P. fici* is similar to that of *F. oxysporum* and *Nectria haematococca*. Interestingly, those fungi (genera *Fusarium* and *Nectria*) and *P. fici* are known to be pathogen on some host plants, but have been isolated as endophytes from others [31].

Our analysis showed an extreme increase in the number of enzymes involved in the degradation of plant cell wall (PCW) oligosaccharides and polysaccharides (Additional file 1: Figure S6). Compared with other sequenced fungi, *P. fici* has a higher number of candidate pectinases and covers all pectinase families known from fungi, including polysaccharide lyase family 1 (PL1), PL3, PL4, PL9, glycoside hydrolase family 28 (GH28), GH78, GH88, GH95, GH105 and GH115 (Additional file 1: Figure S6). The predominant families of pectinases in the *P. fici* genome are PL1 and GH28, having 19 and 22 encoding genes, respectively (Additional file 1: Figure S6). The results of subcellular localization of CAZymes show that almost all the pectinases are secreted (Additional file 2: Table S4). As a component of the vegetal cell wall and the intercellular spaces, pectin might provide nutrient for endophytic fungi.

Chitin deacetylase modules in the carbohydrate esterase family 4 (CE4) can convert surface-exposed chitin into chitosan to avoid host detection [32]. Like the ectomycorrhizal fungus *L. bicolor*, *P. fici* has up to 16 CE4 modules that can benefit the endophyte by reducing its detection by the plant host (Additional file 2: Table S3).

### Expanded transporter gene families

The transportation system is involved in uptake of essential nutrients and ions, excretion of metabolic end products and deleterious substances, and communication between cells and the environment [33]. A total of 1,346 genes encoding transporters were identified in the *P. fici* genome (Additional file 2: Table S5). The average index of expansion estimated by CAFÉ software was higher in the *P. fici* genome (1.75) than in the 13 other analyzed genomes, indicating the significant expansion of this group of genes in *P. fici*.

MFS transporters are involved in the transport of monosaccharides, oligosaccharides, inositols, drugs, amino acids, nucleosides, organophosphate esters, Krebs cycle metabolites, and a large variety of organic and inorganic anions and cations [34]. Compared with the reference fungi, a significant increase in MFS transporters was observed in the *P. fici* genome, and a total of 545 MFS transporter-encoding genes in 23 different families were predicted, accounting for 68% of secondary transporters (Additional file 2: Table S6). The gene number of sugar porter (SP) family of MFS subfamily was higher in the *P. fici* genome (Additional file 2: Table S6), indicating the uptake of more plant-produced nutrients. Comparative analysis with other fungi revealed that the Drug:H^+^ Antiporter-1 (DHA1) and DHA2 family genes are overrepresented in the *P. fici* genome, with 97 and 65 genes, respectively, suggesting export of more metabolism production (Additional file 2: Table S6). The Anion:Cation Symporter (ACS) family had significantly expanded in the *P. fici* genome, i.e., *P. fici* had 144 ACS family genes, that is four times higher than average found in other studied genomes (Additional file 2: Table S6). Of the 144 genes, 65 belong to the Tna1 clade, a high affinity nicotinate permease that catalyzes nicotinic acid (vitamin B3) uptake, reflecting that *P. fici* might be dependent from the host plant for vitamin B3 supply.

### Great biosynthetic capabilities of secondary metabolites in *P. fici*

Secondary metabolites are involved in intracellular, intercellular, and interspecific interactions [35,36]. *Pestalotiopsis fici* produces a wide variety of secondary metabolites, and this motivated us to find the molecular basis of this production by genome sequencing. The average number of core genes related to secondary metabolites synthesis in ascomycetes is only 48 (Table 2). However, we identified 97 core genes related to secondary metabolism including 27 polyketide synthase (PKSs), 12 non-ribosomal peptide synthases (NRPSs), five dimethylallyl tryptophan synthases (DMATs), four putative PKS-like enzymes, 15 putative NRPS-like enzymes, 15 terpenoid synthases (TSs), seven terpenoid cyclases (TCs), seven fatty-acid synthases (FASs) and five PKS-NRPS hybrids (Table 2). Besides the core genes, the tailing genes, regulators, transporters, and other genes that often clustered with the core genes are required for the biosynthesis of secondary metabolites in fungi. The prediction resulted from the combination of SMURF and antiSMASH illustrated that the majority of these core enzymes distributed into 74 secondary metabolite clusters (Additional file 2: Table S7), which is much more than the reference fungi containing an average of 31 gene clusters. Among the 74 gene clusters, 32% contained at least one MFS transporter that might export metabolites out of cell and approximately 24% contained the ‘narrow’-domain TFs Zn(II)2-Cys6 that may regulate the expression of gene clusters.

**Table 2:** **Numbers of core genes involved in secondary metabolism in** ***Pestalotiopsis fici*** **and selected fungi**

	**PKS**	**NRPS**	**DMAT**	**Hybrid**	**PKS-like**	**NRPS-like**	**TS**	**TC**	**FAS**	**Total**
*P. fici*	27	12	5	5	4	15	15	7	7	97
*G. graminicola*	37	5	6	4	1	7	15	4	9	88
*M. oryzae*	27	16	8	2	0	16	16	7	3	95
*F. oxysporum*	9	7	2	2	2	12	13	7	13	67
*F. verticillioides*	11	7	1	2	1	11	16	5	13	67
*F. graminearum*	14	10	0	1	1	11	17	4	8	66
*N. haematococca*	12	8	1	1	1	4	8	5	7	47
*S. sclerotiorum*	15	5	1	0	2	5	7	4	3	42
*V. albo-atrum*	8	6	0	1	2	7	7	5	8	44
*V. dahliae*	7	2	0	1	1	7	12	9	16	55
*A. sarcoides*	2	4	1	0	1	3	9	4	7	31
*E. festucae*	11	18	4	1	3	9	9	4	2	61
*G. lozoyensis*	24	6	2	5	1	13	14	5	6	76
*T. reesei*	11	8	0	2	1	5	11	5	4	47
*N. crassa*	6s	3	1	0	2	3	7	5	4	31
*S. cerevisiae*	0	0	0	0	1	0	4	3	1	9
*T. melanosporum*	2	1	0	0	1	2	5	4	5	20
*L. bicolor*	2	0	1	0	1	2	15	6	4	31
*Pi. indica*	1	0	1	0	0	1	7	4	4	18

PKS, polyketide synthase; NRPS, non-ribosomal peptide synthase; DMAT, dimethylallyl tryptophan synthase; Hybrid, polyketide synthase-non-ribosomal peptide synthase hybrid; TC, terpenoid cyclase; TS, terpenoid synthase and FAS, fatty-acid synthase.

**Figure 5:**
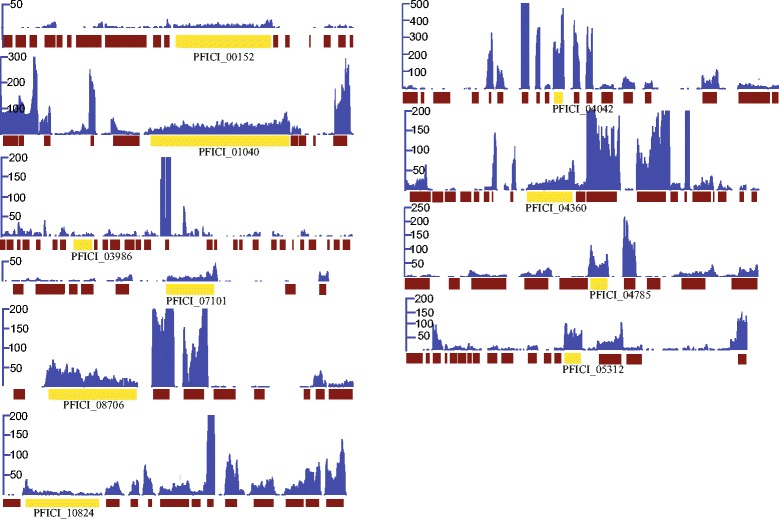
**Visualization of RNA-Seq coverage across the** ***Pestalotiopsis fici*** **secondary metabolite clusters.** The blue curves indicate read coverage for the sample in the rice fermentation medium, the core gene of secondary metabolite was indicated in yellow.

As shown in Figure 5, out of the 74 gene clusters detected in the genome sequence of *P. fici*, only 10 were identified to be active by expression profiling (including one terpene, one NRPS, one NRPS-like, one hybrid NRPS-PKS, six PKSs; and one gene cluster that has been demonstrated to encode for a precursor of chloropupukeanolides: C–E pestheic acid in a concurrent study [37]). Notably, these data, along with the results on the numerous novel secondary metabolites already obtained, indicate the huge potential for the production of secondary metabolites of this fungus.

Fungal PKS genes are mainly type I iterative PKSs (iPKSs) that are further classified into fungal reducing PKSs (RPKSs) and non-reducing PKSs (NRPKSs) based on the degree of reduction in their final products. Although the numbers of PKS genes are similar to those in plant pathogens, such as *Magnaporthe oryzae* (27 genes) and *Glomerella graminicola* (37 genes), PKS genes in *P. fici* are more diverse, including three NRPKS genes, one type III PKS gene (with only a KS domain), a 6-methylsalicylic acid synthase (MSAS) gene, five hybrids of PKS and NRPS, and 24 RPKSs. In addition, PKS domain of PKS-NRPS hybrid is usually followed by NRPS domain in fungal genomes. Interestingly, four among the five PKS-NRPS hybrids from the *P. fici* genome are that NRPS domain is followed by PKS domain (Additional file 1: Figure S7).

The KS domain is the most conserved and can be used to infer the genealogy of the PKS genes. Phylogenetic analysis based on KS domains showed that the *P. fici* proteins grouped in different clusters. One 6-MSAS (PFICI_12928) and four NRPS-PKS hybrid genes (PFICI_04360, PFICI_06351, PFICI_07789, and PFICI_15331) from the *P. fici* genome are nested in the bacterial PKS clade (Additional file 1: Figure S7). Hybrid PKS-NRPS genes PFICI_07941 were grouped with several hybrid PKS-NRPS genes from *M. oryzae* and *G. graminicola* in the subclade IV of RPKS clade, which were composed of a RPKS and a truncated NRPS module. The PKS gene PFICI_00294 was grouped with the lovastatin non-ketide synthase encoding gene MGG_11638T0. The PKS gene PFICI_02353 was grouped with the fumonisins encoding gene FGSG_01790T0, and they shared the same domain structure. In addition, PKS gene PFICI_12549 shared the same domain structure with PFICI_02353. The PKS gene PFICI_07101 was within the melanin pigment group, including the known pigment encoding genes MGG_07219T0 and GLRG_04203. The PKS gene PFICI_06561 shared 59% similarity with the gene FGSG_09182T0 that encodes for biosynthesis of the violet pigment in *F. graminearum.* However, modular analysis showed that PFICI_06561 included a more reducing domain (dehydratase domain). The similarity between PFICI_00149 and PFICI_12888 (40%), PFICI_00366 and PFICI_03986 (46%), PFICI_04360 and PFICI_15331 (59%), and PFICI_07942 and PFICI_15221 (34%) respectively indicated that they were resulted from recent gene duplication.

### Putative genes for the Diels-Alder reaction

The Diels-Alder reaction is the most important step for the transformation in the biosynthesis of cyclohexene-containing secondary metabolites. Diels-Alderases in the prokaryotic actinobacterium *Saccharopolyspora spinosa* have been identified [38]. Although the Diels-Alderases in fungi have not been well documented, several purified enzymes, such as macrophomate synthase [39], have been suggested to involve in the Diels-Alder-type cycloaddition. The *P. fici* genome contained the most putative genes (21) encoding Diels-Alderases, followed by the *Verticillium albo-atrum* genome, with only 10 genes (Additional file 1: Figure S8). Of the 21 putative genes in *P. fici*, 15 were located in gene clusters involved in secondary metabolism. Phylogenetic analysis also revealed that the putative Diels-Alderase genes in *P. fici* were grouped into different clades, suggested that they had higher diversity (Additional file 1: Figure S8).

## Discussion

*Pestalotiopsis fici* genome harbors more multigene families but lacks highly similar paralogs. The genome analysis of *Neurospora crassa* and *F. graminearum* has indicated that the process of RIP, in which duplicated sequences are subject to extensive mutation, may result in the lack of highly duplicated sequences [40,41]. The coexistence of more multigene families and higher RIP in *P. fici* genome supports the viewpoint that gene duplication has occurred before the emergence of RIPs proposed for the *N. crassa* genome [40].

The fungal endophyte-plant host interaction has been hypothesized to be determined by a finely tuned equilibrium between fungal virulence and plant defense [42]. Endophyte-like pathogens possess virulence factors that are countered by plant defense [43]. The gene families involved in detoxification and virulence have undergone expansion in the *P. fici* genome, which may help *P. fici* counter the plant host. CYPs are involved in many essential cellular processes, such as the conversion of hydrophobic intermediates of primary and secondary metabolic pathways and the detoxification of natural and environmental pollutants [44]. The expanded CYPs in the *P. fici* genome mainly participate in primary metabolism, secondary metabolism, defense against host-secreted factors, and xenobiotic metabolism (Additional file 2: Table S8). CYPs also evolve and thereby help fungi adapt to different ecological niches [45]. The CYP57 families, involved in defense against host secreting factors, had also undergone expansion in the *P. fici* genome (Additional file 2: Table S8). The high diversity of secondary metabolites is related to the diversity of the CYP genes. For example, the 219 CYP genes in the *Ganoderma lucidum* genome resulted in a large number of different secondary metabolites [46]. The CYP families in *P. fici* associated with secondary metabolism such as CYP58, 59, 65, 67, 503, 530, 532, 536, and 537 had undergone significant expansion.

The CAZymes analysis provides useful information about fungal life strategies [47]. Though it lacks experimental supports, the numbers of CAZymes seem to relate to the nutritional availability [48] and lifestyle of fungi associated with plant. Obligate parasitic fungi deriving nutrients from living tissues have the fewest CAZymes [49,50], followed by biotrophic pathogens, symbiotic fungi such as *L. bicolor* and *Tuber melanosporum* have fewer CAZymes [51-53]. The saprotrophic fungi have fewer CAZymes than plant pathogenic fungi, especially lacking families involved in degrading living plant tissues, because they can obtain nutrients from plant residues. Compared with obligate biotrophic plant pathogen and symbiotic fungi, necrotrophic and hemibiotrophic plant pathogens have relatively more CAZymes [48], because those fungi have relatively limited nutrients within plant tissue. The fungi with dual lifestyles as endophyte and pathogen have high diversity and number of CAZymes because those fungi should adapt to endophytic lifestyle to utilize the limited intercellular nutrients from plant tissue. Pectin is the major component between cells of the living plant tissues. The expansion of pectinase putative genes in *P. fici* genome provides more evidence for its endophytic lifestyle.

Transporters involved in uptaking nutrients from plants have undergone significant expansion in bacterial endophytes [54]. The higher number of SP family genes in *P. fici* indicates an enhanced capacity for uptaking limited carbohydrates from plants. The expansion of Tna1 clade belonging to ACS family suggests that *P. fici* might be dependent from host for vitamin B3 supply. MFS transporters from DHA1 family and DHA2 family are able to export drugs to the environment [33]. Consistent with abundant transporters from DHA1 family and DHA2 family, export of more metabolites facilitates that *P. fici* communicates with host plant.

Fungi interact with other organisms and environment factors in their living niches. Endophytic lifestyle is one of many factors that affect capacity of fungal secondary metabolites, and not all endophytes are rich in secondary metabolite production. Compared with the endophytic *Ascocoryne sarcoides*, *Epichloë festucae*, and *Pi. indica*, *P. fici* genome showed abundant secondary metabolites and a high diversity of core enzyme-encoding genes and gene clusters for secondary metabolites. However, the transcriptional profile indicated that only a few of these gene clusters are expressed under certain culture condition. Although many gene clusters may be cryptic when *P. fici* is growing *in vitro*, the environment influences their secondary metabolites *in planta* considering the fact that endophytes reside within plants and are interacting with their hosts. The co-culture of an endophytic fungus with its host plant cells *in vitro* may enhance the production of fungal secondary metabolism and promote discovery of novel natural products.

The NRPS/PKS hybrids in Dothideomycetes, Eurotiomycetes, and Sordariomycetes have been acquired from bacteria via horizontal gene transfer (HGT) in the relatively early evolution of the Pezizomycotina [55]. Our phylogenetic analyses of PKS genes revealed the bacterial origination of four NRPS/PKS hybrids in *P. fici* genome via HGT. This result was also supported by the NRPS/PKS hybrid PFICI_06351 which does not contain introns. However, another three hybrid genes PFICI_04360, PFICI_07789, and PFICI_15331 contain seven, two, and eight introns, respectively. These results may be explained by the divergence time of those genes. Appearance and evolution of introns in the genes acquired from bacteria remains unknown and need further investigation. In addition, a 6-MSAS gene (PFICI_12928) in *P. fici* was also apparently from bacterium via HGT. Therefore, HGT could be one major approach for the diversity generating and maintaining of PKS genes in *P. fici*.

The gene duplication is the second approach and may be more important than HGT for generating PKS gene diversity in *N. crassa* [56]. Genome analysis of *P. fici* revealed that four pairs of paralogous PKS genes (PFICI_00149 and PFICI_12888, PFICI_00366 and PFICI_03986, PFICI_04360 and PFICI_15331, and PFICI_07942 and PFICI_15221) may be generated by duplications. Although high RIP process in the *P. fici* genome may result in the lack of highly duplicated sequences, gene duplication has occurred before the emergence of RIPs. Overall, the diversity of PKSs in the *P. fici* genome may result from both gene duplication and HGT.

## Conclusions

In conclusion, we report on the genome sequencing, comparative genome analysis, and transcriptional analysis of secondary metabolite clusters in endophytic fungus *P. fici* of tea (W106-1/CGMCC3.15140). The predicted gene clusters of secondary metabolism obviously enhance the identification of biosynthesis pathway of known compounds, and show the huge potential for drug discovery from natural products of *P. fici*. Besides, the sequence data also offer a better understanding of life strategy of plant endophyte *P. fici*, namely that abundance of extracellular pectinase adapts to lifestyle of living tissue of plant and uses pectin as nutrient. The genome sequence will facilitate future studies into mining novel bioactive secondary metabolites of plant endophyte and plant-endophyte interactions.

## Methods

### Organism and the reference genomes

*Pestalotiopsis fici* (W106-1/CGMCC3.15140) is isolated from branches of *Camellia sinensis* in the suburb of Hangzhou, China. Chemical investigation shows that it is prolific producer of bioactive secondary metabolites [40]. 17 fungal genomes were used to compare with the *P. fici* genome. The detail information of these genomes was listed in Additional file 2: Table S1.

### Transformation of GFP-tagged *P. fici* and microscopy

A binary vector pKS 2251 (kindly provided by Professor Seogchan Kang, Department of Plant Pathology and Environmental Microbiology, Pennsylvania State University) containing a hygromycin resistance gene and the green fluorescent protein (GFP) gene was transformed into *P. fici* (W106-1/CGMCC3.15140)*.* Transformants expressing GFP were selected under ultraviolet light with a Zeiss Axio imager A1 microscope. The living tea trees were collected from Eshan County, Yunnan province and grew in greenhouse in Beijing. The twigs of the living tea trees were inoculated with the transformant expressing GFP. Seven and 21 days after inoculation, optical sections of infected plant material were collected and analyzed using a Leica TCS-SP2 confocal microscope. GFP fluorescence was detected with a 515 nm bandpass emission filter and autofluorescence of the plant cell walls was detected with a 595 nm bandpass emission filter.

### Genome sequencing and assembly

*Pestalotiopsis fici* (W106-1/CGMCC3.15140) was sequenced using a whole-genome shotgun sequencing approach at the Chinese National Human Genome Center (Shanghai, China). Three runs of Roche 454 GS FLX standard pyrosequencing generated 2,999,862 reads (a 24.5-fold sequence depth). The reads were first assembled using Newbler software Version 2.3, which produced 586 contigs. Then a DNA library of 3-kb inserts was constructed and sequenced on an Illumina/Solexa Genome analyzer using a paired-end module to construct the scaffolds. SSPACE and GapFiller software was conducted to further fill the gap and generate scaffolds. The data has been deposited at DDBJ/EMBL/GenBank under accession: ARNU00000000.

### Gene prediction and genome annotation

The *P. fici* genome was annotated using fungal/eukaryotic genome annotation pipeline of Broad Institute [57]. The gene structures were predicted using a combination of several gene predictors: 1) *Ab Initio* predictors GeneMark-ES [58], GENEID [59], FGENESH [60], Augustus [61] and GlimmerHMM [62]; 2) homology-based predictors GENEWISE [63], and TBLASTN against UniRef90 nonredundant protein dataset [64]; 3) PASA alignment assemblies [65] and Transcript Reconstruction. The parameter of GENEID is the foxysporum file. *Fusarium graminearum* is used as the training set of Augustus. Then the predicted gene modelers were combined into consensus gene structure annotations using EvidenceModeler [66]. The gene product names are assigned by BLAST against SwissProt, Superfamily and by HMMER against Pfam [67,68], TIGRfam [69]. Automated functional annotation was performed using protein sequences deduced from all gene models automatically predicted. The protein domains were identified using InterProScan [70] which runs a set of methods including pattern matching and motif recognition. In addition, we used an automated assignment against protein domain databases such as GO [71], KEGG [72], KOG [73], and FUNCAT [74]. Three criteria were used to support the gene calls. The first based on identification of functional domains of PFAM database [68]. The second based on identification of orthology to genes in other fungi using OrthoMCL [75]. The third relied on expression data obtained from Illumina Solexa sequences, and the RNA-seq was seen below.

### Transposable elements (TE) and repeat-induced point mutations (RIP)

TEs were identified in the *P. fici* genome *de novo* using RepeatScout with the default parameters (l = 15) to generate libraries of consensus sequences [76]. These libraries were then filtered as follows: all sequences shorter than 200 base pairs were discarded and repeats with fewer than 10 copies were removed. The remaining consensus sequences were annotated manually by tBLASTx against Repbase [77]. *De novo* repeats were mapped to the genome using RepeatMasker [78], then the number of TE occurrences and the percentage genome coverage were assessed. The repeat families were aligned via ClustalW version 2.0.12, and the RIP index was calculated using RIPCAL [79].

### Multigene families and evolutionary analysis of protein families

Multigene families were generated from proteins in *P. fici* and in other sequenced reference fungi (Additional file 2: Table S1) by orthoMCL using the default parameters, except for the inflation parameter [75]. Inflation parameter 1.5 was used for the clustering procedure and the proteins were organized into 13,752 protein families. Of those, 8,238 families contained at least one *P. fici* protein and 140 protein families, containing 358 proteins, were specific to the *P. fici* genome.

Evolutionary changes in protein families were analyzed using CAFÉ version 2.2 [80]. All the protein families from the MCL analysis were used to identify change of protein families. In total, 11,012 protein families were used in the CAFÉ analysis after exclusion of unique proteins families. Based on 122 single-copy orthologous genes from the *P. fici* and other reference fungi (Additional file 2: Table S1), a phylogenetic tree was constructed using the parallelized version of RAxML 7.2.8 with the PROTGAMMAJTT model with 100 rapid bootstrap replications [81]. To estimate the divergence times, the RAxML tree was used to apply a penalized likelihood analysis in the program r8s v1.7 [82] with the origin of the Ascomycota at 500-650 mya [83].

The mean size and standard deviation for all the gene families (excluding orphans and lineage-specific families) were calculated. The counts by species for each family were transformed into a matrix of *z-*scores so that the data could be centered and normalized. The 105 families with the greatest *z-*score in *P. fici* were hierarchically clustered using Pearson’s correlation, and clustering and visualization were performed using MeV software. The biological function of each family was predicted using the PFAM database [68] and the FunCat database [74].

### Targeted annotation and analysis of specific gene families

The detection and determination of module composition and family assignments of all carbohydrate-active enzymes (CAZymes) was performed as described for the CAZy database using the dbCAN HMMER-based classification system [84]. Biclustering of GH families and organisms was performed using R [85]. Genes encoding transporters were annotated by BLASTP using transporter encoding genes retrieved from the Transport Classification database with a cut-off of *E*value1e-20. Lineage-specific gene expansion and contraction were estimated using the CAFÉ software [80].

### Analysis of core genes and gene clusters involved in secondary metabolism

The web-based prediction tool SMURF and the antiSMASH pipeline were used to predict secondary metabolic gene clusters and core genes [86,87]. The genes encoding terpenoid synthases, terpenoid cyclases, fatty-acid synthases were identified using the Superfamily database. Then the core genes were manually curated using the PFAM database [68].

### Assignment of catalytic domains of PKS genes and KS domain genealogy construction

Domains were manually assigned by referencing computational predictions using a combination of the Management and Analysis for Polyketide Synthase Type I, ITERDB [88], and the Conserved Domain Database (CDD) from the NCBI. The PKS types were determined using domain composition and the available literature [89] and included hybrids of PKS and NRPS, bacterial iPKS (bMSAS or bPRPKS), 6-MSAS, NRPKS, PRPKS, and RPKS. Using the predicted KS domains of *P. fici*, other reference fungi and outgroups of the homologous FASs from animals and representative type I PKSs from bacteria (Listed in Additional file 2: Table S9) were aligned by MAFFT6.717b [90]. Then RAxML protein trees were produced for the protein alignments using the PROTGAMMAJTT model with 100 rapid bootstrap replications [81]. The tree and domain compositions were visualized using iTOL [91].

### Identification of and phylogenetic analysis of putative Diels-Alderases

The solanapyrone synthase gene (Alternaria_SOL5, accession number: AB514562) has been reported as possible Diels-Alderases that was applied as a query to blast against the protein sequences of *P. fici* [92]*.* A total of 21 putative Diels-Alderases genes were identified in the *P. fici* genome and grouped into two homologous groups. The sequences of all homologous genes from the two homologous groups in the *P. fici* genome and other reference genomes were aligned by MAFFT6.717b [90]. Then RAxML protein trees were produced for the protein alignments using the PROTGAMMAJTT model with 1000 rapid bootstrap replications [81].

### Transcriptome analysis

In order to utilize transcriptional data to define the secondary metabolites clusters, a time course experiment was conducted on rice as substrate on which abundant secondary metabolites were detected in previous study. They were sampled at five-day intervals for a total of eight time points (days 5, 10, 15, 20, 25, 30, 35, and 40), then analyzed by LC-MS. Natural products were reached to the peak after 20 days. The total RNA from the time point days 20 was extracted with TriZol® according to the manufacturers protocol (Invitrogen). Messenger RNA was purified and after reverse transcription into cDNA, the libraries were constructed according to the massively parallel signature protocol [93]. Then they were sequenced with Illumina technique. The RNA-seq reads were mapped to the genome with Tophat [94]. The RNA-seq data were visualized with the IGB-browser [95] and the gene cluster was considered to be expressed if the mRNAs of the core genes in the gene cluster were detected. The RNA-seq expression dataset is available at the NCBI’s expression Omnibus under the accession code GSE60046.

### Aviailability of supporting data

This Whole Genome Shotgun project has been deposited at DDBJ/EMBL/GenBank under the accession ARNU00000000. The version described in this paper is the first version, ARNU01000000. The RNA-seq expression dataset has been deposited at the NCBI’s Gene Expression Omnibus under the accession code GSE60046. The phylogenic alignments have been deposited in TreeBase; submission ID 17070, (http://purl.org/phylo/treebase/phylows/study/TB2:S17070?x-access-code=437ed86497182c431809582dbe80bf9&format=html).

## Additional files

Additional file 1:
**Supplemental figures.** This document contains Supplemental Figures S1 to S8 and their legends. Additional file 2:
**Supplemental tables.** This file contains Supplemental Tables S1 to S9.

## Abbreviations

Mb: Mega base pairs
TE: Transposable element
RIP: Repeat-induced point mutation
PL: Polysaccharide lyase
GH: Glycoside hydrolase
CE: Carbohydrate esterase
GT: Glycosyl transferase
CBM: Carbohydrate-binding module
CFEM: Cysteine-rich fungal-specific extracellular EGF-like
MFS: Major facilitator superfamily
PCW: Plant cell wall
SP: Sugar porter
DHA: Drug:H+ Antiporter
ACS: Anion:Cation Symporter
PKS: Polyketide synthase
NRPS: Non-ribosomal peptide synthase
DMAT: Dimthylallyl tryptophan synthase
TS: Terpenoid synthase
TC: Terpenoid cyclase
FAS: Fatty-acid synthase
iPKS: iterative polyketide synthase
MSAS: 6-methylsalicylic acid synthase
RPKSs: Reducing PKSs
NRPKSs: Non-reducing PKSs
HGT: Horizontal gene transfer
CYPs: Cytochrome P450 monooxygenases
