# Supplementary material for: Genomic and transcriptomic analysis of the endophytic fungus Pestalotiopsis fici reveals its lifestyle and high potential for synthesis of natural products
Source: BMC Genomics. 2015 Jan 27;16(1):28. doi: 10.1186/s12864-014-1190-9 (PMC4320822; doi:10.1186/s12864-014-1190-9)
Supplement: Additional file 1: — Supplemental figures. This document contains Supplemental Figures S1 to S8 and their legends. [file 12864_2014_1190_MOESM1_ESM.zip › Supplement figure legends.docx]

**Supplemental Figure legends:**

**Figure S1 Biotrophic growth of *Pestalotiopsis fici* in a tea branch.** A–C, longitudinal sections of a tea branch 21 days after inoculation with *P. fici* hyphae; A, fluorescent micrograph of tea and hyphae; B, brightfield micrograph of A; C, overlay of fluorescent and brightfield micrographs; D–F, cross sections of a tea branch 21 days after inoculation with *P. fici* hyphae; D, tea branch and hyphae; E, brightfield micrograph of D; F, overlay of fluorescent and brightfield micrographs. Scale bar = 100 μm.

**Figure S2 Evidence for gene prediction accuracy in *Pestalotiopsis fici*.** Three types of evidences were combined: (i) Functional annotation (at least one domain/motif), (ii) Orthologous genes between *P*. *fici* and selected fungi, (iii) Solexa RNA-sequence support.

**Figure S3 Fold changes in di-nucleotide abundances for all repeat families of *Pestalotiopsis fici* compared to the nonrepetitive control sequence on a Log_10_ scale.**

**Figure S4 Gene count per OrthoMCL family for each species. Genes were clustered into families using OrthoMCL software**. The x-axis bins indicate the number of genes per family, while the counts of y-axis indicate the total number of genes in each bin per species.

**Figure S5 Hierarchical clustering of the top 105 *Z-scores* for *Pestalotiopsis fici* gene family sizes.** The *Z-scores* scale indicates that the gene family size is smaller (blue) or the larger (red). So, the red blocks reflect gene family expansions. The gene families were constructed by OrthoMCL. Left: Gene family size in different sizes. Right: Gene family description (Pfam families); function annotation (FunCat).

**Figure S6 Biclustering of glycoside hydrolase gene families and polysaccharide lyase gene families in *Pestalotiopsis fici* and other selected fungi.** In the top tree, the fungi are abbreviated as follows: *Pestalotiopsis fici* (PF), *Verticillium albo-atrum* (VA), *Magnaporthe oryzae* (MO), *Epichloë festucae* (EF), *Fusarium graminearum* (FG)*, Glomerella graminicola* (GG), *Sclerotinia sclerotiorum* (SS), *Neurospora crassa* (NC), *Tuber melanosporum* (TM), *Ascocoryne sarcoides* (AS), *Piriformospora indica* (PI), *Saccharomyces* *cerevisiae* (SC), *Laccaria bicolor* (LB) and *Trichoderma reesei* (TR). The left tree shows the class number of the enzyme families according to the carbohydrate-active enzyme database. The values in the matrix indicate the number of gene encoding enzymes detected for each combination of enzyme family and fungus. A color scale from white (0 occurrences) to red (42 occurrences) indicates the abundance of the different enzymes per species.

**Figure S7 Phylogenetic tree of polyketide synthases (PKS) inferred by maximum likelihood analysis of the KS domain and the domain architecture of PKS proteins**. The accession numbers of the bacterial PKS genes (Bact) and the fatty acid synthase genes from animals (Fas) obtained from GenBank are shown in Table S9. Scale bar represents the number of substitutions per site. The KS number is indicated by protein IDs, as follows: PFICI: *Pestalotiopsis fici*, VDBG, *Verticillium albo-atrum*; MGG, *Magnaporthe oryzae*; EFQ, *Epichloë festucae*; FGSG, *Fusarium graminearum*; GSTUM, *Glomerella graminicola*; NCU, *Neurospora crassa*; sarcoides, *Ascocoryne sarcoides* and Trire2, *Trichoderma reesei*. The domain architecture of the proteins is shown on the right. Protein domain names are as follows: KS, ketoacyl synthase; AT, acyltransferase domain; ACP, acyl carrier domain; KR, ketoreductase; DH, dehydratase; ER, enoyl reductase; TD, terminal domain; TE, thioesterase; ME, methyl transferase and PP, 4′-phosphopantetheinyl transferase; C, condensation domain; AMP, adenylation domain; PCP, peptidyl carrier protein. Support values below 40% are not shown.

**Figure S8 *Pestalotiopsis fici*-specific expansion of genes that may encode Diels-Alderase.** The fungi names are *Pestalotiopsis fici* (PFICI), *Verticillium albo-atrum* (VDBG), *Magnaporthe oryzae* (MGG, black color), *Epichloë festucae* (EFQ), *Fusarium graminearum* (FGSG), *Glomerella graminicola* (GSTUM).
