# Supplementary material for: Genomic and transcriptomic analysis of the endophytic fungus Pestalotiopsis fici reveals its lifestyle and high potential for synthesis of natural products
Source: BMC Genomics. 2015 Jan 27;16(1):28. doi: 10.1186/s12864-014-1190-9 (PMC4320822; doi:10.1186/s12864-014-1190-9)
Supplement: Additional file 1: — Supplemental figures. This document contains Supplemental Figures S1 to S8 and their legends. [file 12864_2014_1190_MOESM1_ESM.zip › S4.pdf]

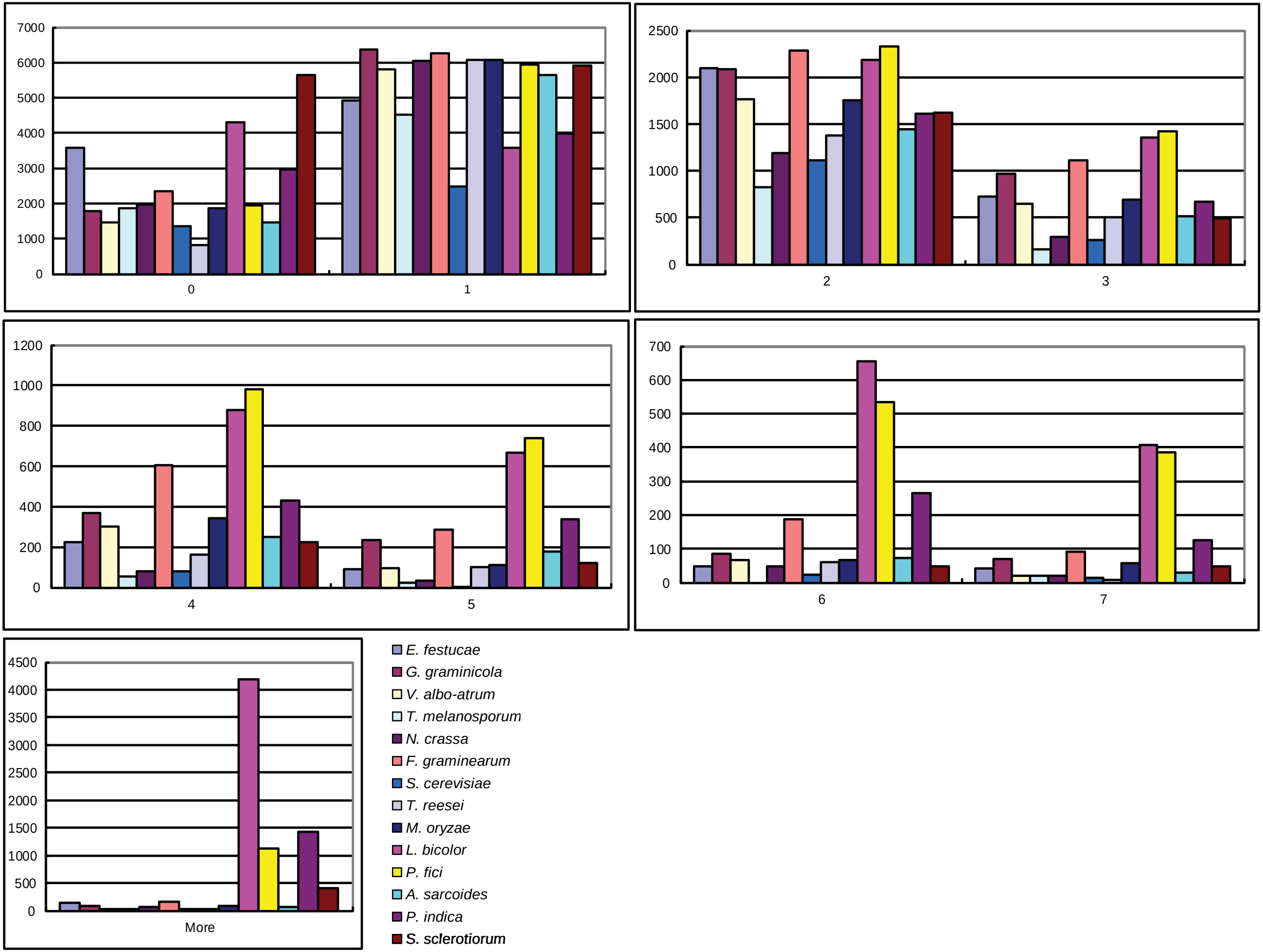
7000
6000
5000
4000
3000
2000
1000
0
0
1
2500
2000
1500
1000
500
0
2
3
1200
1000
800
600
400
200
0
4
5
700
600
500
400
300
200
100
0
6
7
4500
4000
3500
3000
2500
2000
1500
1000
500
0
More
E. festucae
G. graminicola
V. albo-atrum
T. melanosporum
N. crassa
F. graminearum
S. cerevisiae
T. reesei
M. oryzae
L. bicolor
P. fici
A. sarcoides
P. indica
S. sclerotiorum
