# Supplementary figures and images for: Genomic and transcriptomic analysis of the endophytic fungus Pestalotiopsis fici reveals its lifestyle and high potential for synthesis of natural products
Source: BMC Genomics. 2015 Jan 27;16(1):28. doi: 10.1186/s12864-014-1190-9 (PMC4320822; doi:10.1186/s12864-014-1190-9)

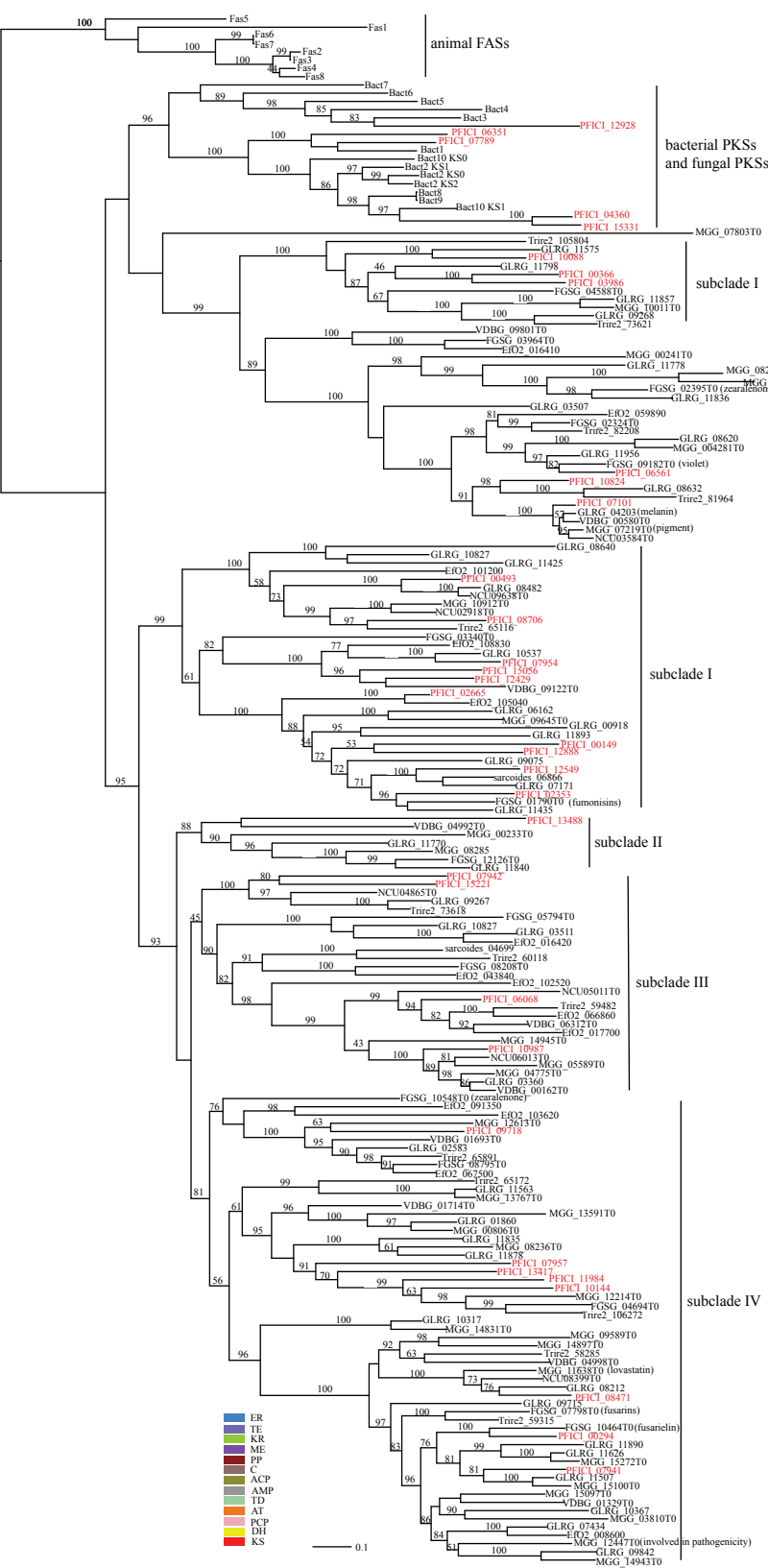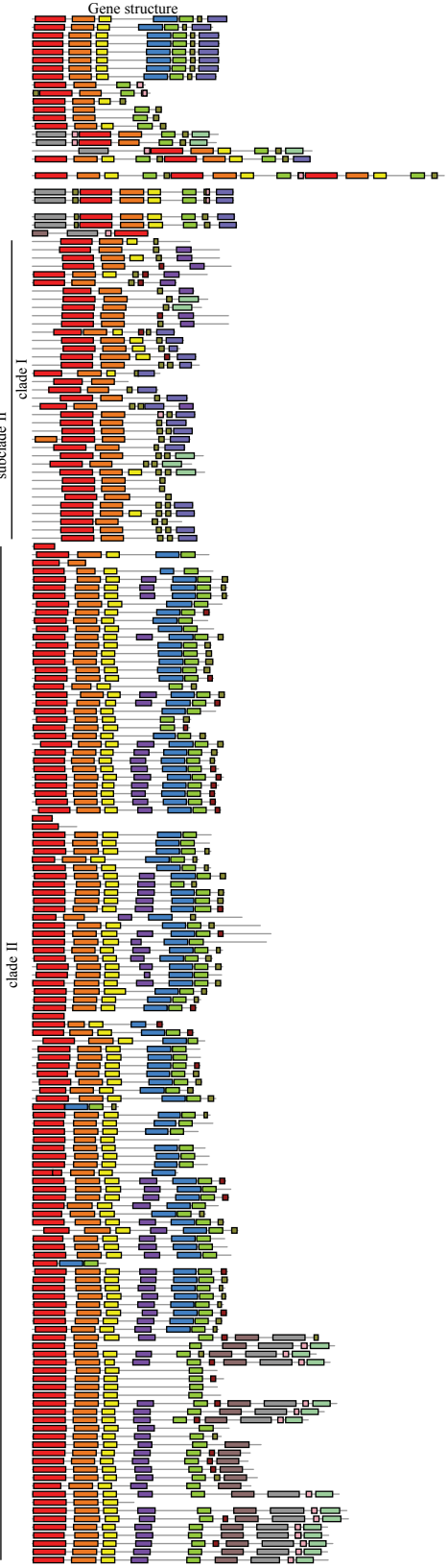

Supplement: Additional file 1: — Supplemental figures. This document contains Supplemental Figures S1 to S8 and their legends. [file 12864_2014_1190_MOESM1_ESM.zip › S7.pdf]

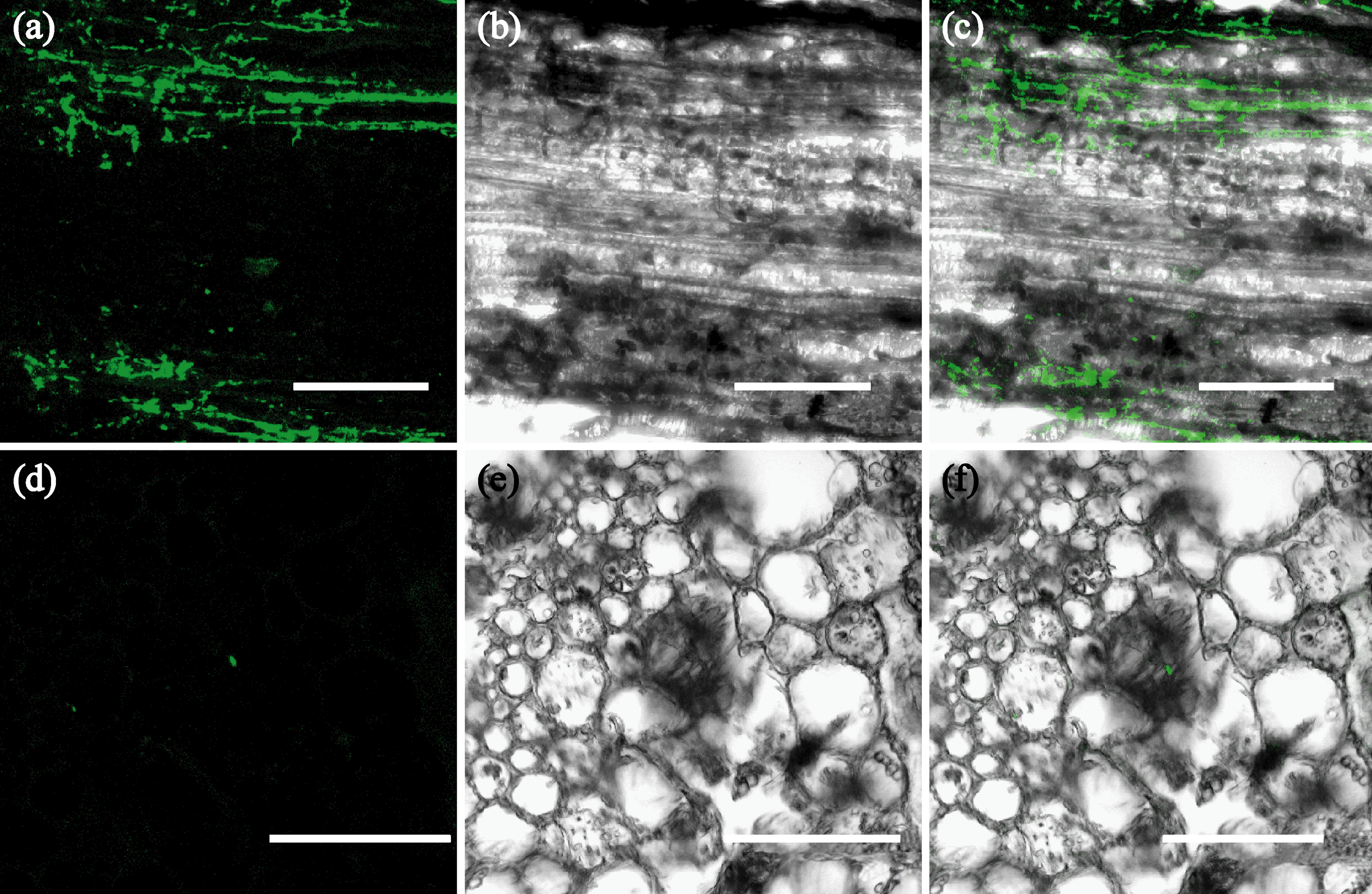

Supplement: Additional file 1: — Supplemental figures. This document contains Supplemental Figures S1 to S8 and their legends. [file 12864_2014_1190_MOESM1_ESM.zip › S1.tif]

**Functional evidence**  
(14,528 genes)  
(94%)

**Orthology evidence**  
(11,755 genes)  
(76%)

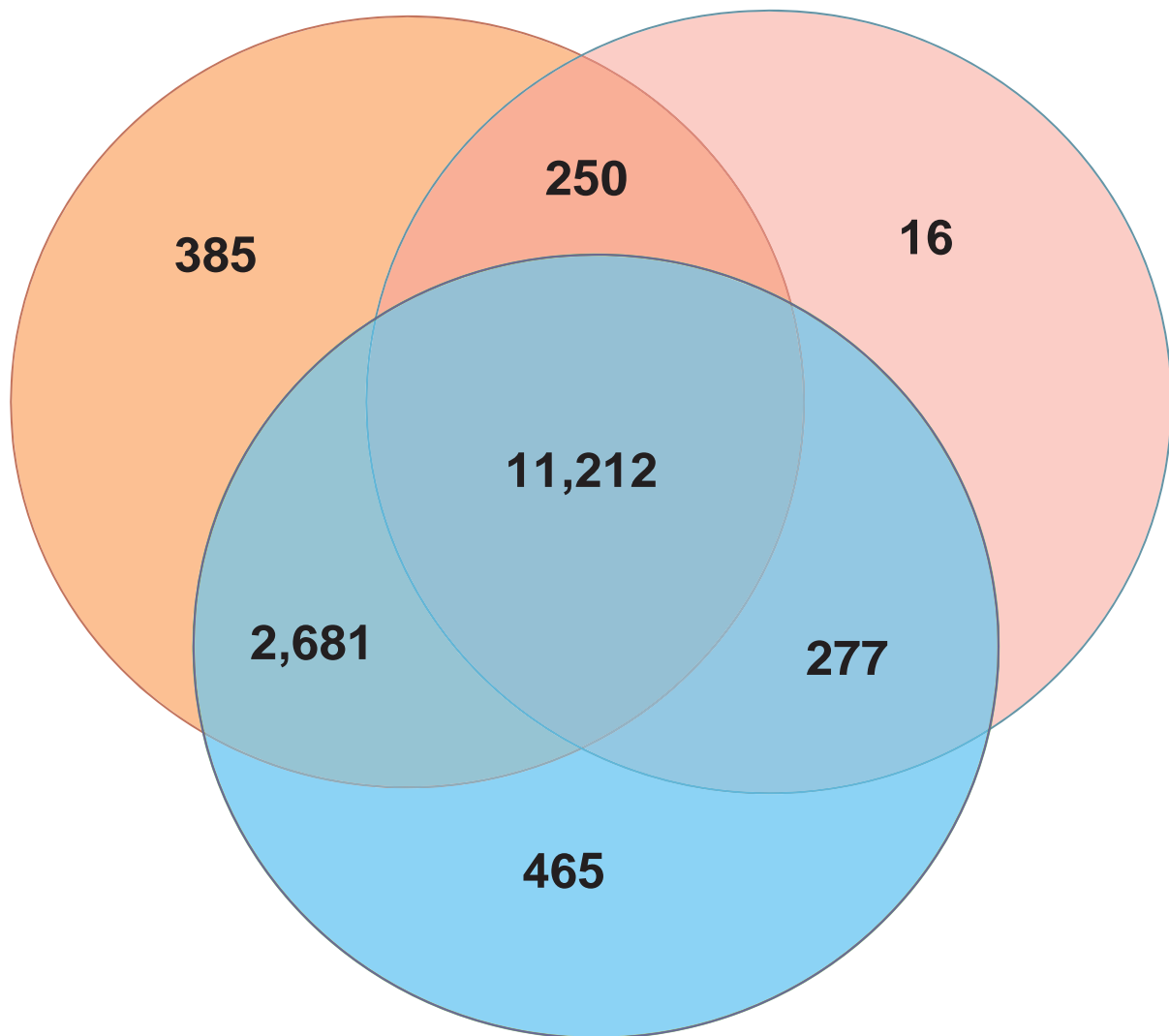

**RNA-seq evidence**  
(14,635 genes)  
(95%)

Supplement: Additional file 1: — Supplemental figures. This document contains Supplemental Figures S1 to S8 and their legends. [file 12864_2014_1190_MOESM1_ESM.zip › s2.pdf]

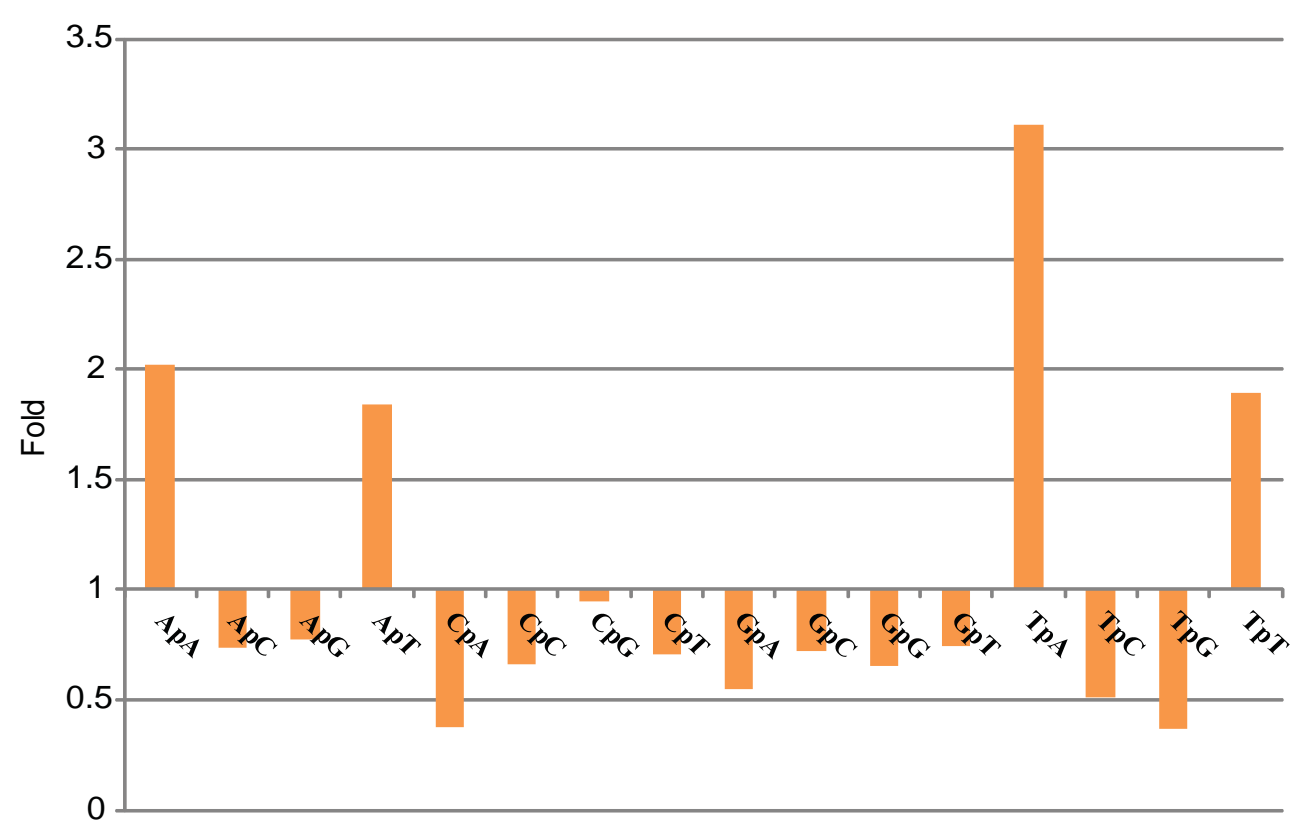

Supplement: Additional file 1: — Supplemental figures. This document contains Supplemental Figures S1 to S8 and their legends. [file 12864_2014_1190_MOESM1_ESM.zip › S3.pdf]

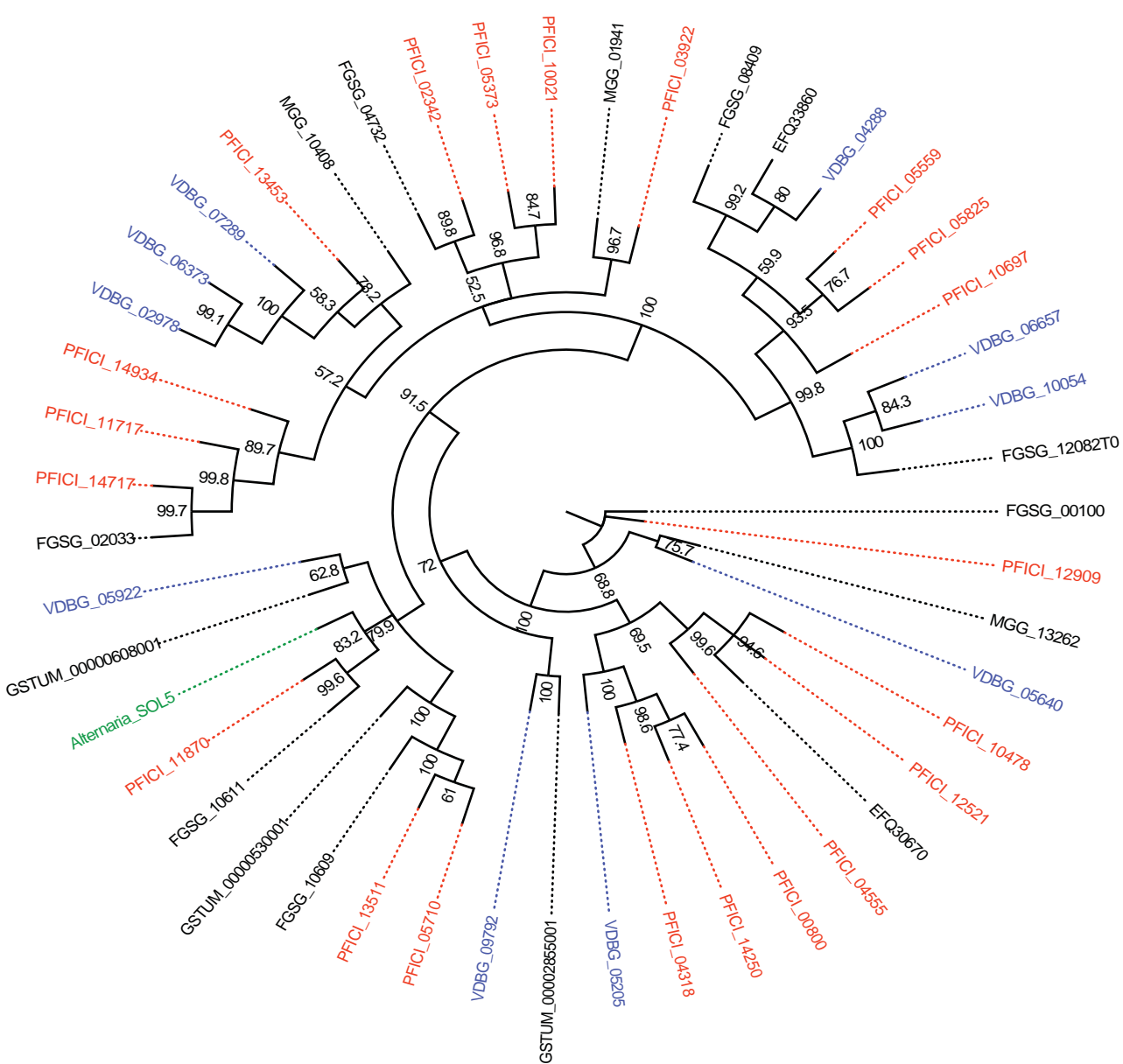

Supplement: Additional file 1: — Supplemental figures. This document contains Supplemental Figures S1 to S8 and their legends. [file 12864_2014_1190_MOESM1_ESM.zip › S8.pdf]

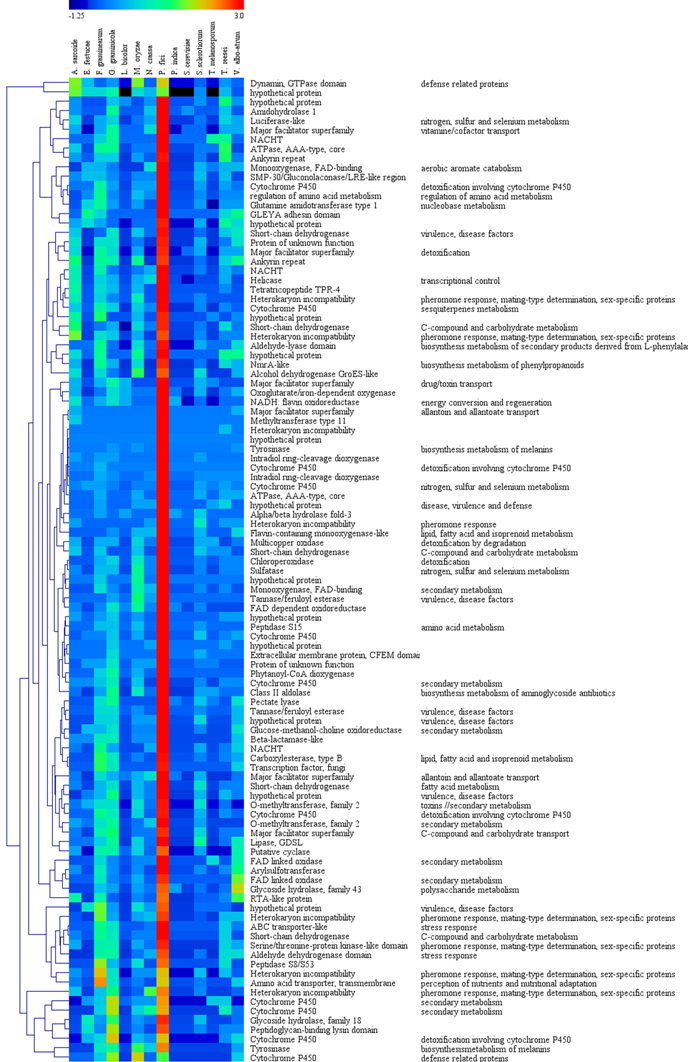

Supplement: Additional file 1: — Supplemental figures. This document contains Supplemental Figures S1 to S8 and their legends. [file 12864_2014_1190_MOESM1_ESM.zip › S5.tif]

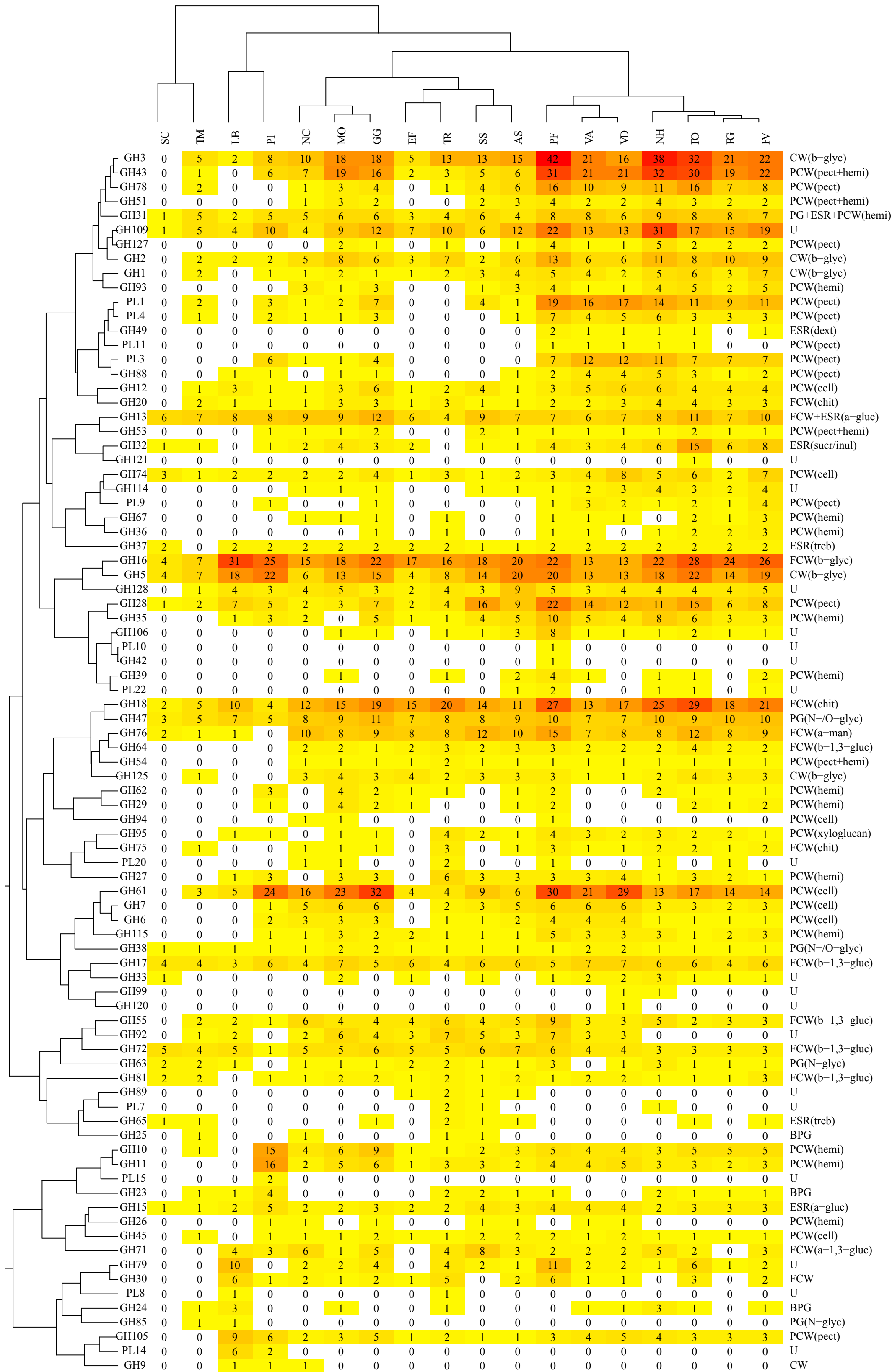

Supplement: Additional file 1: — Supplemental figures. This document contains Supplemental Figures S1 to S8 and their legends. [file 12864_2014_1190_MOESM1_ESM.zip › S6.pdf]
